# Deconvolving the contributions of cell-type heterogeneity on cortical gene expression

**DOI:** 10.1371/journal.pcbi.1008120

**Published:** 2020-08-17

**Authors:** Ellis Patrick, Mariko Taga, Ayla Ergun, Bernard Ng, William Casazza, Maria Cimpean, Christina Yung, Julie A. Schneider, David A. Bennett, Chris Gaiteri, Philip L. De Jager, Elizabeth M. Bradshaw, Sara Mostafavi

**Affiliations:** 1 School of Mathematics and Statistics, The University of Sydney, Sydney, New South Wales, Australia; 2 The Westmead Institute for Medical Research, The University of Sydney, Sydney, New South Wales, Australia; 3 Center for Translational & Computational Neuroimmunology, Department of Neurology, Columbia University Medical Center, New York City, New York, United States of America; 4 Research and Development, Biogen, Cambridge, Massachusetts, United States of America; 5 Departments of Statistics and Medical Genetics, University of British Columbia, Vancouver, British Columbia, Canada; 6 Centre for Molecular Medicine and Therapeutics, Vancouver, British Columbia, Canada; 7 The Bioinformatics Training Program, University of British Columbia, Vancouver, Canada; 8 Department of Pediatrics, Division of Rheumatology, Washington University School of Medicine, St. Louis, Missouri, United States of America; 9 Rush Alzheimer’s Disease Center, Rush University Medical Center, Chicago, Illinois, United States of America; 10 Department of Neurology, Columbia University Medical Center, New York City, New York, United States of America; University of California Irvine, UNITED STATES

## Abstract

Complexity of cell-type composition has created much skepticism surrounding the interpretation of bulk tissue transcriptomic studies. Recent studies have shown that deconvolution algorithms can be applied to computationally estimate cell-type proportions from gene expression data of bulk blood samples, but their performance when applied to brain tissue is unclear. Here, we have generated an immunohistochemistry (IHC) dataset for five major cell-types from brain tissue of 70 individuals, who also have bulk cortical gene expression data. With the IHC data as the benchmark, this resource enables quantitative assessment of deconvolution algorithms for brain tissue. We apply existing deconvolution algorithms to brain tissue by using marker sets derived from human brain single cell and cell-sorted RNA-seq data. We show that these algorithms can indeed produce informative estimates of constituent cell-type proportions. In fact, neuronal subpopulations can also be estimated from bulk brain tissue samples. Further, we show that including the cell-type proportion estimates as confounding factors is important for reducing false associations between Alzheimer’s disease phenotypes and gene expression. Lastly, we demonstrate that using more accurate marker sets can substantially improve statistical power in detecting cell-type specific expression quantitative trait loci (eQTLs).

## Author summary

Gene expression data generated from a tissue sample reflects an average gene expression profile across heterogeneous populations of cells. Because composition of constituent cell-types can vary across individuals (due to technical or biological factors), differential gene expression analysis requires estimating and adjusting for such cellular heterogeneity. While many *deconvolution algorithms* for estimating cellular composition from tissue gene expression data have been tested extensively in blood, their performance when applied to brain tissue is unclear. To address this gap, we generated an immunohistochemistry (IHC) dataset for five major cell-types from brain, in order to apply and then assess deconvolution algorithms for application to brain gene expression datasets. We show that these algorithms can indeed produce informative estimates of constituent cell-type proportions. Further, we show that adjusting for estimated cell-type proportions across individuals when conducting differential gene expression analysis is important in reducing false associations.

## Introduction

Understanding the molecular aetiology of neurodegeneration and neuropsychiatric diseases holds the promise of developing safe and effective treatments which despite decades of work are still lacking. Large collaborative efforts such as the CommonMind Consortium[1] and the AMP-AD venture[2] have been constructed to address these unmet needs. These consortia have deeply phenotyped the brains of thousands of individuals with an array of ‘omic technologies including RNA and DNA sequencing. The vast majority of this molecular profiling has been performed on bulk tissue samples meaning that changes in the measured expression of a gene can be due to altered gene activity or simply because there has been a change in the number of cells that express that gene. Hence while promising, the complex cell-type composition of the brain creates a level of uncertainty around the interpretation and validity of reported molecular-disease associations, including our own[3], which demands further investigation.

Observed gene expression levels in tissues with high cellular heterogeneity are influenced by the proliferation or death of specific cell-types and also by molecular processes within cell-types. In the context of disease studies, this ambiguity in the origin of gene expression variability can generate spurious disease associations or reduce statistical power to detect true associations[4]. Separating out the contributions of cell-type composition on gene expression, through a mathematical method known as deconvolution, should result in more accurate disease associations.

Cell-type deconvolution methods are a spectrum of analytical approaches for separating out changes in gene expression stemming from shifts in cell-type compositions from alterations in gene activity. Typically variations of factor analysis and regression[5], deconvolution approaches use known cell-type specific genes to generate robust estimates of cell-type composition. Modelling these changes in cell-type composition not only facilities segregating changes in gene expression associated with cell-type changes from those associated with disease-associated activity but can also be used to infer which cell-types these changes in activity are occurring. This potential has been experimentally validated in specific settings, for instance on immune cell subsets[6].

A key contribution to the reliability of deconvolution approaches are the cell-type markers, or genes that are expressed predominately in a given cell-type, that are used in the modelling. Recent single-cell RNA-seq[7–9] and cell-sorted datasets[10] from human brain tissue can enhance the effectiveness of deconvolution methods through more accurate identification of cell-type marker genes. Deconvolution algorithms are being applied to gene expression in the brain using these cell markers to infer and adjust for glial cell subsets with higher granularity[11–13]. However, because of lack of availability of high-resolution benchmark datasets across multiple individuals, their accuracy and resolution is not well understood.

In this work, we constructed a benchmark brain dataset for quantitative evaluation of deconvolution algorithms. Specifically, we generated an immunohistochemistry (IHC) imaging dataset and quantified the proportions of five distinct cell-types from the cortex of 70 individuals, for which bulk-tissue RNA-seq data have also been acquired. With the IHC dataset as the benchmark, we evaluated four state-of-the-art deconvolution algorithms whose effectiveness has not been assessed in brain tissue. We applied these algorithms to brain tissues by using three sets of marker genes derived from human brain single-cell RNA-seq data[7], human brain cell-sorted RNA-seq data[10], and cell-sorted microarray data[12]. We also explored whether proportion of neuronal subpopulations can be estimated from bulk brain tissue samples. Further, we assessed the importance of including cell-type proportion estimates as confounding factors when associating gene expression to disease phenotypes. Lastly, we tested the cell-type proportion estimates for detecting cell-type specific expression quantitative loci (eQTL) in the brain. The IHC data generated as part of this study, as we all code for all analysis and results is available from https://github.com/ellispatrick/CortexCellDeconv.

## Results

### Generation and quantification of IHC data

To establish a benchmark for cell-type proportions in heterogamous brain tissue, we used immunohistochemistry to experimentally measure the proportion of neurons, astrocytes, microglia, oligodendrocytes, and endothelial cells from dorsolateral prefrontal cortex (DLPFC) tissue of 70 older individuals. These individuals are a subset of the larger ROSMAP cohort with bulk RNA-seq (n = 508) from the same region[3]; donors showed a range of cognitive function, from healthy to Alzheimer’s dementia (e.g., 33% incident of Alzheimer’s dementia; **S1 Table**), which likely enhances the heterogeneity of cell-type proportions (**S1 Fig**).

**Fig 1:**
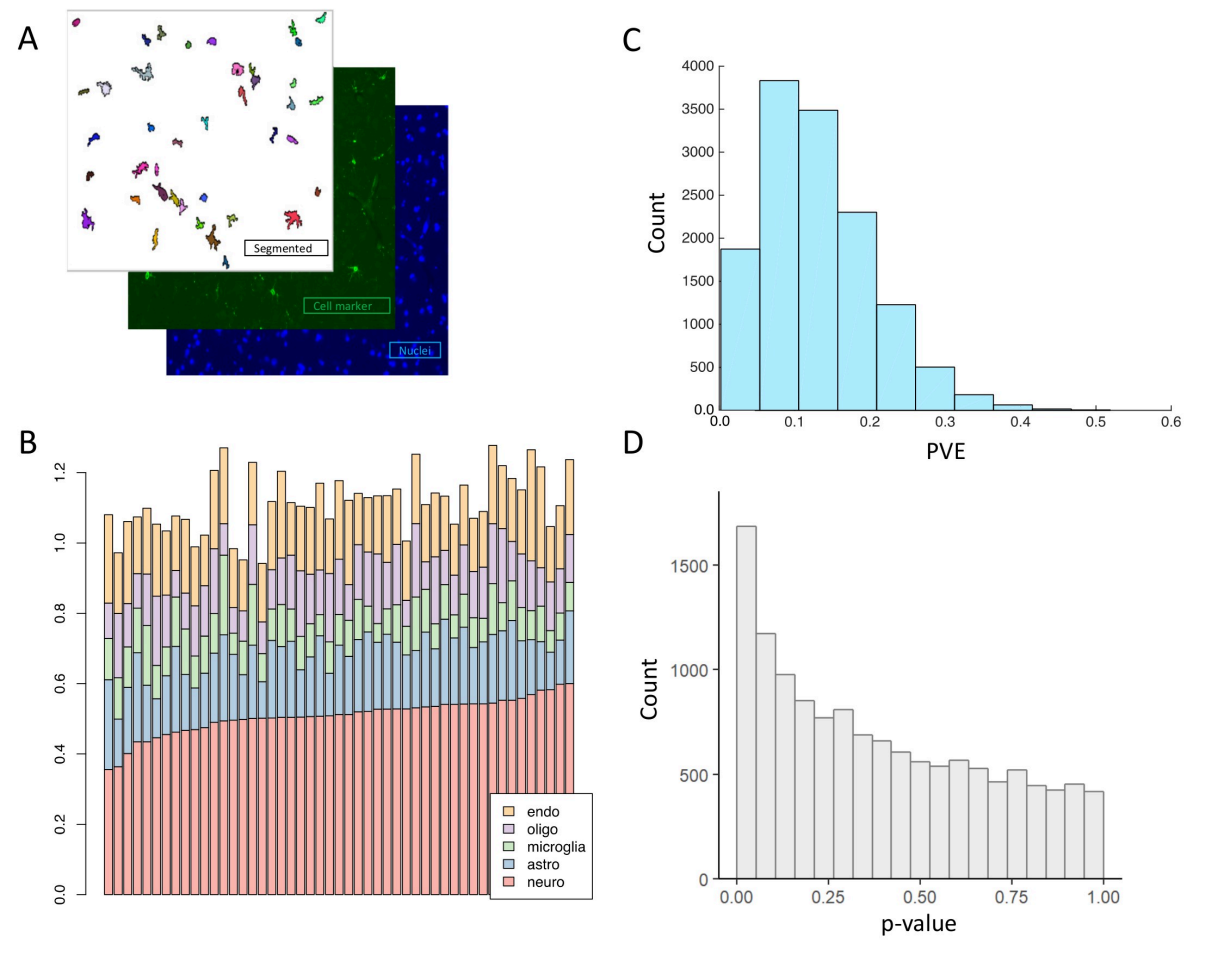
Estimation of cell-type proportions by IHC. (A) Figure depicts an example segmented IHC image used to quantify cell-type proportions. (B) A bar plot illustrating the total proportions of cell-types for an individual. Each bar represents an individual, y-axis shows the estimated proportion of each of the five cell-types. The proportions of the different cell-types for a specific individual are estimated from different images. The sum of the proportions of the cell-types should be close to one. (C) A histogram showing the Percent Variance Explained (PVE) of expression values of all genes (across 70 individuals) by the combination of proportion of five cell-types measured by IHC. For each gene, linear regression was used to estimate the gene expression levels of that gene across individuals from five covariates (representing IHC proportions from each of the five cell-types). A value of one would mean that all the variation in a gene’s expression could be explained by the IHC estimated proportions of cell-types. All values are less than 0.6. (D) A p-value distribution corresponding to the PVE histogram in panel C, showing the p-values for the correlation between gene expression levels (all expressed genes) and IHC-based cell-type proportions estimates across 70 individuals with paired data. As in panel C, expression level of each gene was used as the outcome in linear regression, with covariates included for IHC measurements from each cell-type. A peak at zero provides evidence that the variation of many genes can be explained by changes in cell-type proportions.

To generate IHC-based cell-type proportions, antibodies were chosen to identify neurons (NeuN), astrocytes (*GFAP*), microglia (*IBA1*), oligodendrocytes (*OLIG2*), and endothelial cells (*PECAM*). Automated image analysis (EBImage) was applied to identify DAPI stained cells and the cells that were positive for each antibody (**Fig 1A**). The proportion of each cell-type was derived by averaging 30 images taken from 6 μm slides per individual. Confirming the quality of the IHC-based cell-type proportions, we observed that the proportion of the five major cell populations per subject approximately sums to one, despite separate staining performed for each cell-type (**Fig 1B**). That is, because each cell-type is stained and counted independently, both natural and technical variability (counting error or staining efficacy) can result in the sum of the proportions being greater or less than one. The sum of the cell-types approximately reaching one also implies that the five measured cell-types make up the bulk of the DLPFC, with no other major cell population unmeasured.

We find that variability in the ROSMAP gene expression can be explained by changes in cell-type proportions. We previously derived cell-type specific modules of covarying genes from this cohort[3] which we claimed mimicked the behavior of cell-type proportion shifts. Encouragingly. the expression levels of these gene modules correlate with the IHC estimates of cell-type proportions. (**S2 Fig**). Moreover, when looking at global gene expression levels, the IHC-based cell-type proportions explain ~11% of the variance in gene expression levels (**Fig 1C**) and are found to correlate with the expression levels of a large number of genes (**Fig 1D**). These results confirm that cell-type heterogeneity is indeed a major contributor to the variation in bulk-tissue gene expression data from the brain, also verifying that our expression data is a relevant test bed for evaluating deconvolution algorithms.

### Adapting and evaluating deconvolution algorithms

Recent studies have shown that cell-type proportions in blood samples can be reliably estimated through computational means, but the accuracy of these estimates for brain cell-type deconvolution is currently unclear. To establish the validity of computational estimation of cell-type proportions in brain tissue, we used the IHC data as the benchmark to assess the accuracy of four deconvolution methods. The methods fall into two classes: 1) “supervised” reference-based methods, which included non-negative least squares (NNLS)[14], CIBERSORT[15], and dtangle[5], and 2) “semi-supervised” reference-based, exemplified by the digital sorting algorithm (DSA)[16]. Both classes rely on pre-defined marker genes (also referred to as signature gene lists) for each cell-type derived from reference profiles. The distinction is that supervised approaches also require cell-type specific expression profiles of the marker genes as derived from cell-type specific gene expression datasets.

To adapt these deconvolution algorithms to brain tissue, we examined 3 sets of cell-type marker genes derived from: (1) human brain single-cell RNA-seq data (“Zhang”), (2) human brain cell-sorted RNA-seq data[7] (“Darmanis”), and (3) a curated collection of cell-sorted microarray data and In-Situ Hybridization from mouse brains (Neuroexpresso)[12]. For each data source, differential gene expression analysis identified sets of marker genes that are preferentially expressed in each of the five cell-types (see Methods).

We assessed the concordance between the IHC-based cell-type proportions and estimates generated by the deconvolution algorithms with two metrics: (1) correlation and (2) mean squared error (MSE) between the inferred and measured proportions for each cell-type across individuals. With correlation, we assess whether individuals with higher proportion estimates also display higher proportion in the IHC data, i.e. the *relative* proportions across individuals is assessed, but not the *absolute* values of the proportions. Having high correlation is often adequate for the estimated proportions to be useful for downstream analysis; for example when estimates are used as confounding factors in association analysis, only the accuracy of relative proportions is important. On the other hand, if the abundance of different cell-types in varied brain regions is of interest, the *absolute* difference between the estimated and ground truth proportions as measured with MSE is more appropriate.

**Fig 2:**
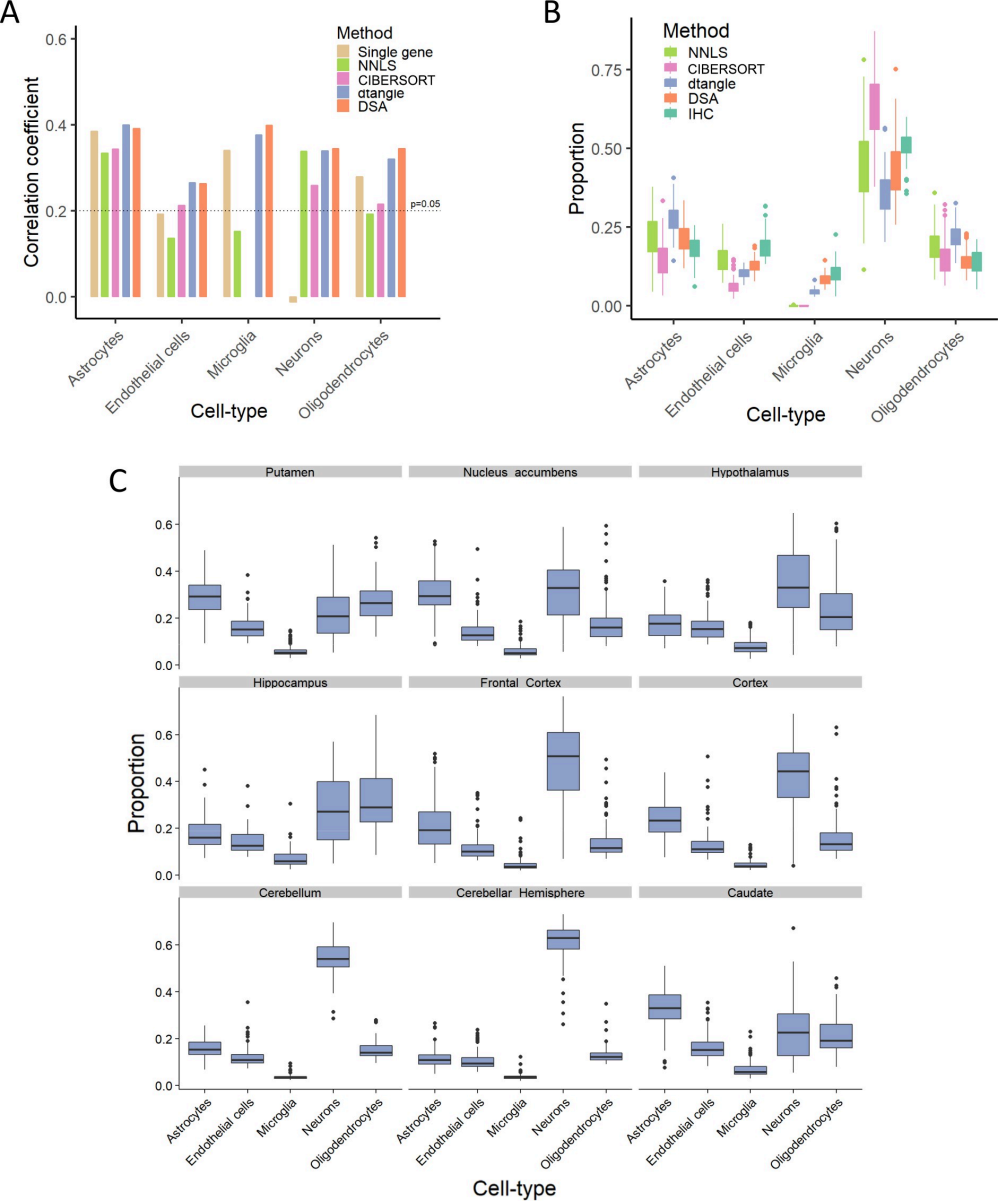
Computational estimation of cell-type proportions. (A) Figure shows the Spearman correlation coefficient between IHC-based cell-type estimates and four deconvolution algorithms, in addition to the “single marker” based approach. For the single marker based approach, we used the expression of the widely used marker genes: ENO2 for neurons, GFAP for astrocytes, CD68 for microglia, CD34 for endothelial, OLIG2 for oligodendrocytes. Correlations larger than 0.2 provide evidence that the gene expression cell-type proportion estimate for that cell-type are correlated with the IHC cell-type proportion using an unadjusted p-value threshold of 0.05. (B) Estimates of absolute proportions of each cell-type in the DLPFC according to the four algorithms tested, and IHC (experimentally measured in this study). Box plots depict the range of proportions across 70 individuals. (C) Boxplots depict the similarities and differences of predicted cell-type proportions (using DSA algorithm and Zhang markers) across nine brain regions, based on bulk GTEx tissue data.

We observed that the correlations between IHC and deconvolution estimates are mostly significant, with moderate effect sizes, but variable results for endothelial cell proportions (**Figs 2A** and **S3**). We also observed that the various algorithmic approaches yield highly correlated estimates as assessed more robustly across a larger set of 508 ROSMAP samples (**S4 Fig**). However, CIBERSORT and NNLS are “outliers” in this respect for estimation of microglia cells, which might stem from their difficulty in estimating such low abundant cell-type (**Fig 2A**). Overall, correlation results are similar for the different sources of marker genes (**S5 Fig**), and so we report the results with “Zhang” markers in the main text and “Darmanis” markers in the supplement. We note that we found removing technical confounds from gene expression data generally improves the accuracy of the tested algorithms (see **S6 Fig**) which provides evidence that there may be other technical factors such as sample quality or effectiveness of image analysis algorithms that are contributing to the moderate effect sizes.

Although statistically significant, the magnitude of the correlations are only moderate and could have been reduced by technical variability. To approximate upper bounds for the correlation values, we constructed “artificial mixtures” from a brain single cell RNA-seq dataset[17]. Without accounting for noise in image analysis, we estimated the upper bounds to be between 0.5 to 0.7 (**S7 Fig**). The observed correlations between IHC and deconvolution estimates are thus reasonably within the expected range if noise in image analysis is considered.

In addition to evaluating the proportion estimates for each cell-type independently, we also assessed the estimates based on relative magnitude across cell-types. When averaged across samples, the relative magnitude across cell-types show high concordance between the IHC and deconvolution estimates (**Fig 2B**), and are consistent with estimates based on single cell data from cortex[8, 9]. This concordance implies that the estimated proportions are not confounded by the variability in the total amount of RNA across different cell-types, as one may suspect. We also assessed the robustness of these results with respect to variability in the size of marker gene set and found results to be robust for a wide range of sizes (**S8 Fig)**. To test the generalizability of the results, we further estimated cell-type proportions across nine brain regions from GTEx data[18]. For Cortex, we observed highly concordant proportions between estimates derived from the ROSMAP and GTEx datasets (**Fig 2B and 2C**). For other brain regions, we observed strong variations in the estimated cell-type proportions, with adjacent regions tending to yield similar proportions (**Fig 2C**), which demonstrates the stability of the computational estimations. Although not much is conclusively known about the variation in cell-type proportions across human brain regions, encouragingly, these estimates matched what was expected based on cell counts using a single-cell RNA-seq dataset[8] (**S9 Fig**).

In terms of MSE, which is a much stricter criterion for evaluation, we observed greater differences in performance between methods (**S10 Fig**). DSA was the only algorithm that provided significantly accurate absolute proportions for 3 out of 5 cell-types, as assessed by permutation tests (**S10C and S10D Fig**), and interestingly single cell markers (“Darmanis”) generally yielded more accurate absolute proportions (**S10A and S10B Fig**).

### Deconvolution of bulk gene expression data using single-nuclei RNA sequencing profiles

**Fig 3:**
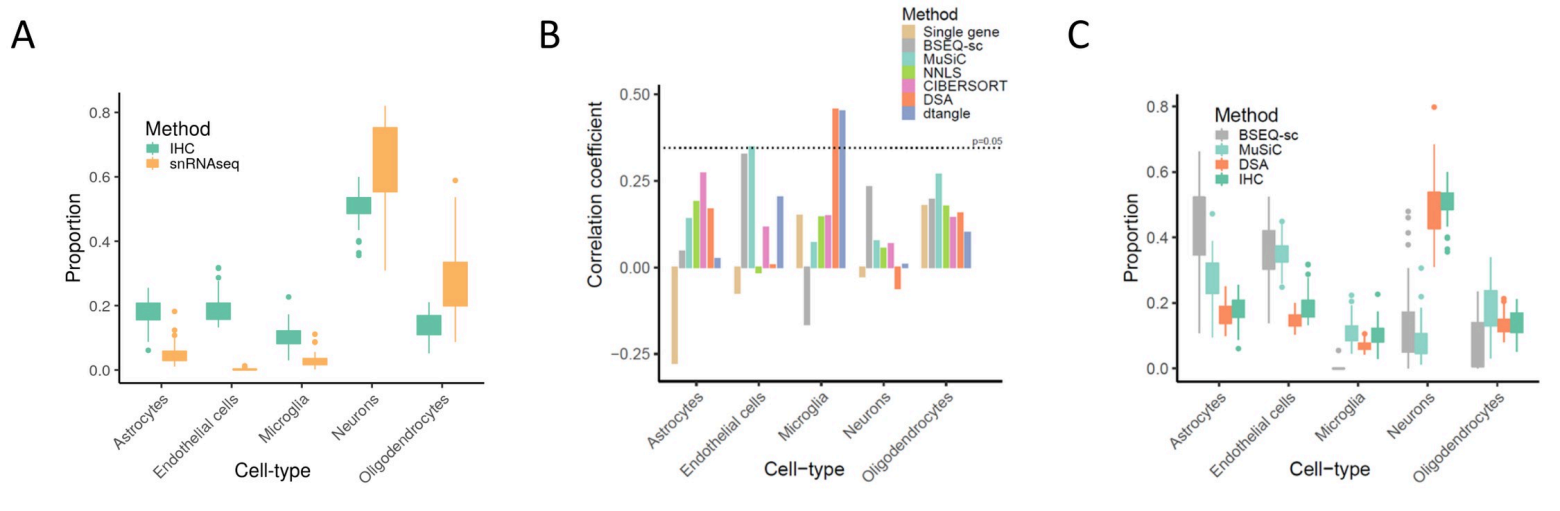
Cell-type proportions estimates with snRNA-seq. (A) Boxplots show the cell-type proportions calculated from our IHC data and cell-type proportions calculated using a snRNA-seq dataset that was also generated from the ROSMAP cohort. Boxplots depict the range of proportions across 48 individuals. The boxplots for each cell-type should substantially overlap if the estimates from both datasets were similar. (B) Barplots of the correlations between the ROSMAP snRNA-seq data and the four deconvolution methods, single gene markers and two additional deconvolution approaches MuSiC and BSEQ-sc. MuSiC and BSEQ-sc are two methods that use snRNA-seq data as a reference to deconvolute bulk gene expression data and here they are using the ROSMAP snRNA-seq data as a reference to deconvolute the ROSMAP bulk gene expression data. These estimates are then compared back to the ROSMAP snRNA-seq proportions. (C) Boxplots depict the predicted proportion of cell-types estimated using MuSiC and BSEQ-sc compared to DSA and IHC. Both MuSiC and BSEQ-sc use cell-type markers and other information from the snRNA-seq data to deconvolute the bulk gene expression data. DSA was chosen to represent other deconvolution approaches as DSA, dtangle, CIBERSORT and NNLS all had similar estimates in Fig 2.

Cell-type proportions estimated using single-nuclei RNA Sequencing (snRNA-seq) data perform poorly in this study. Single-nuclei RNA Sequencing has previously been performed on 48 individuals from the ROSMAP cohort[9] which provides the dual opportunity to compare proportions estimated with deconvolution to proportions calculated directly with the snRNA-seq data and those estimated using the snRNA-seq gene expression profiles as a reference. Average cell-type proportions measured by counting the cell-type calls in the snRNA-seq are substantially different to those calculated by counting in the IHC dataset (**Fig 3A**). While neurons are the most abundant cell-type in each assay, there are very few endothelial cells labelled in the snRNA-seq data. There are also very few astrocytes relative to oligodendrocytes. While this comparison does not make it clear which assay is more accurately identifying proportions, unlike the IHC data, none of the deconvolution algorithms are significantly correlated with the snRNA-seq proportions (**Fig 3B**). Additionally, MuSiC[19] and BSEQ-sc[20] are two deconvolution algorithms that were both designed to use the gene expression profiles from single-cell sequencing to deconvolute bulk expression data. While both methods perform reasonably when estimating relative proportions (**S11 Fig**) neither method estimates absolute proportions well (**Fig 3C**) suggesting that either the snRNA-seq data needs more cleaning, the cell markers used need to be optimized or the snRNA-seq is simply not appropriate for deconvolution in this case. These results indicate that this particular snRNA-seq dataset is not as useful for calculating proportions or informing specialized deconvolution algorithms for estimating proportions in the ROSMAP cohort.

### Inferring neuronal sub-type proportions from bulk gene expression

**Fig 4:**
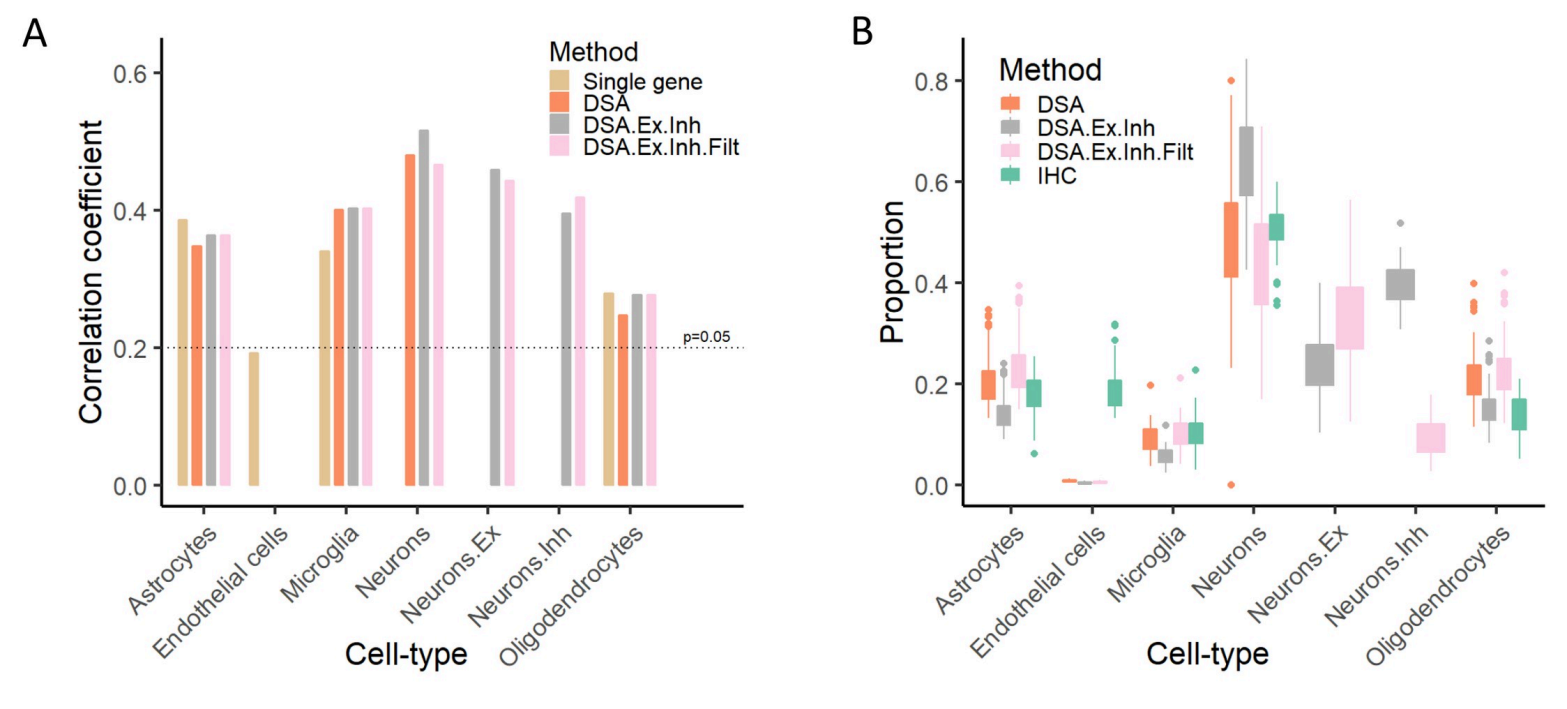
Inference of neuronal sub-types. We used markers for inhibitory and excitatory neurons from Darmanis dataset, to predict the proportion of these two-neuronal sub-types, in addition to oligodendrocytes, endothelial, microglia, and astrocytes. To ensure that the deconvolution algorithms can robustly infer sub-types, we also filtered the list of markers to only include those that are differentially expressed in neurons (and are not also highly expressed elsewhere). (A) correlation between proportions of four major cell-types, in addition to two neuronal-subtypes, with measured IHC data. (B) Inferred proportions for four major cell-types, in addition to two neuronal sub-types. DSA method with Darmanis markers was used. DSA: algorithm was run on five major cell-type, as Fig 2. DSA.Ex.Inh: algorithm was run using four major cell-types, in addition to two neuronal sub-types. DSA.Ex.Inh.Filt: the neuronal sub-type markers where filtered to only include those that are highly expressed in neurons (based on Zhang dataset). Neuron.Ex and Neuron.Inh are the excitatory and inhibitory neurons respectively while, for DSA.Ex.Inh and DSA.Ex.Inh.Filt, Neuron is the sum of these two subsets. If DSA is robust, introducing extra cell sub-types shouldn’t alter the proportion estimates of other cell-types.

Single cell and single nuclei transcriptomic studies of the brain have revealed notable heterogeneity in neurons, with a dozen different neuronal sub-types and states identified based on clustering of gene expression data[9]. Although the IHC data generated as part of this study do not enable direct assessment of the accuracy by which neuronal sub-types can be estimated from bulk-tissue data, we indirectly tested this possibility in two ways. First, we assessed the impact of including excitatory and inhibitory neuronal sub-type markers on the accuracy of the other cell-type proportion estimates. We observed that including neuronal sub-types does not negatively impact the accuracy of the other four major cell-types (**Fig 4A**), so long as genes that span multiple marker sets are filtered out (**S12 Fig**; supplementary methods). Second, we compared the neuronal sub-type proportions with the overall proportion of neurons, and found that the relative proportion of excitatory to inhibitory neurons is consistent with prior reports[9], and the summation of these two sub-types yields proportions similar to that of neurons (**Fig 4B**). Overall, these results suggest that estimation of excitatory and inhibitory neurons is feasible from bulk-tissue RNA-seq. However, the availability of robust markers with cell-type specific expression is critical for accurate inference.

### Using inferred cell-type proportions in association analyses

**Fig 5:**
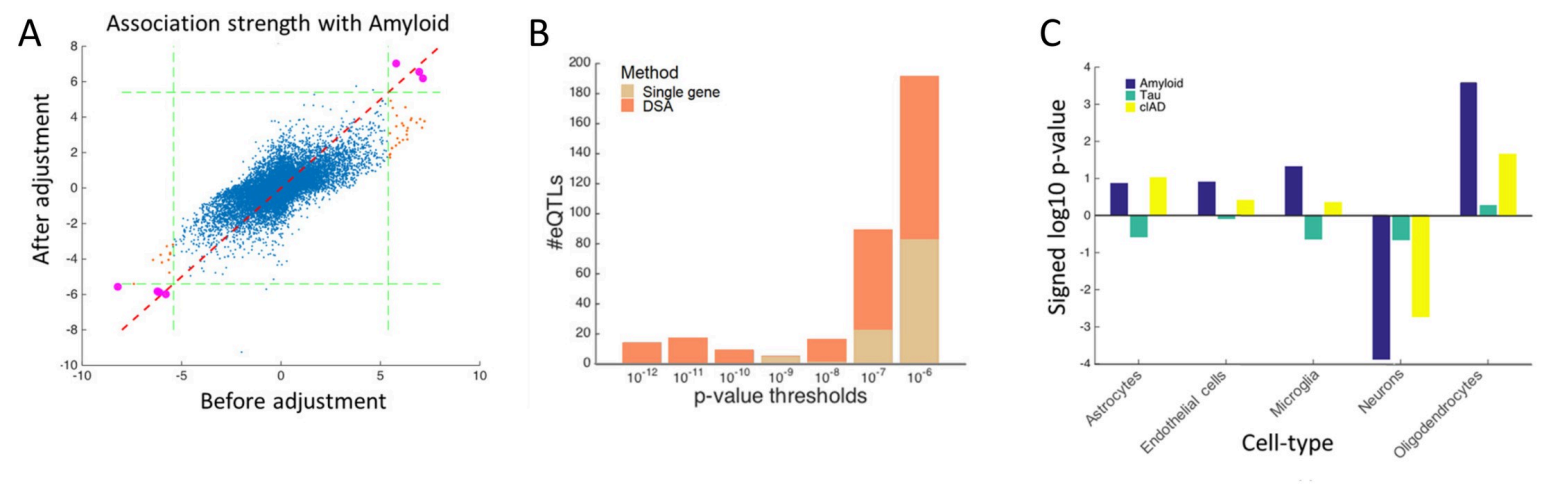
Utility of inferred proportions in association analysis. (A) A scatter plot shows the signed p-value for association between each gene’s expression level and amyloid aggregation, as assessed on the ROSMAP dataset (N = 508). x-axis shows the association strength before adjusting for cellular heterogeneity, and y-axis show the association strength after adjusting for cellular heterogeneity. The dashed green lines mark the Bonferroni corrected p-value threshold based on this signed log p-value representation. The purple dots represent genes that are found to be significant in both adjusted and not-adjusted data; the red dots are genes that are only significant in not-adjusted data. (B) A bar plot shows the signed log10 p-values for association between inferred proportions and three AD related phenotypes. Predictions from DSA across 508 samples were used. (C) Figure shows the number of associations for several p-value thresholds. We tested ~34 x 10^6^ eQTLs in total across cell-types, so the most stringent threshold based on Bonferroni correction is in the 10^−9^ range. We opted to clip the plot at a relaxed range of p < 10^−6^ to better display the differences in performance between using single gene marker sets and multiple gene marker sets. The p-values displayed are raw p-values without multiple testing correction. Number of associations found based on the DSA estimates are shown in blue, and those based on single cell marker genes are shown in yellow.

To assess the relevance of the estimated cell-type proportions in disease studies, we re-analyzed the ROSMAP dataset to identify genes whose expression levels are associated with Alzheimer’s disease (AD) and its related neuropathology, namely amyloid beta and tau proteins. By including the estimated proportions as confounding factors, we observed substantial reduction in the number of genes associated with amyloid beta (**Fig 5A)**, suggesting that the genes found without adjusting for cellular heterogeneity are likely false positives since their variance can be significantly explained away by variability in cell-type proportions. Supporting this, we observed significant correlations between amyloid levels and proportions of oligodendrocytes and neurons (**Fig 5B**). Similar trend, but to a lesser degree, was observed for association with clinical AD.

In addition to using the estimated cell-type proportions to correct for cellular heterogeneity in disease studies, another important application of these estimates is the discovery of cell-type specific genetic regulation of gene expression. Recent blood-based studies have shown the feasibility of inferring cell-type specific eQTLs from bulk-tissue gene expression data, so long as accurate estimates of cell-type proportions are available. To demonstrate the feasibility of this approach for brain, we performed cell-type-specific eQTL analysis[21] with the estimated cell-type proportions. We hypothesized that deconvolution algorithms that use multi-gene marker sets should yield more accurate estimates of cell-type proportions, and hence increases the statistical power for detecting cell-type specific eQTLs compared to using single gene markers, which indeed is the case (**Fig 5C**).

## Discussion

Here we addressed the apprehension surrounding interpretation of gene expression measurements from post-mortem brain tissue by demonstrating that existing deconvolution algorithms can be used for estimating cell-type proportions in the brain. The application of these algorithms enable better utilization of the large number of existing well-annotated bulk post-mortem RNA-seq datasets to study brain-related disease and gene regulation.

The benchmark dataset that we produced provides a resource that can be used to further optimize cell-type deconvolution algorithms for use in brain tissue. Cell-type deconvolution algorithms have predominately been developed and evaluated in whole blood, PBMC and tumors[6], where samples are easily obtained and already in suspension. Brain tissue is complex, needs to be dissociated and is typically obtained post-mortem which can also affect the measurement of gene expression[22]. As such, our report of the performance of deconvolution algorithms is encouraging, demonstrating that they can be used and providing a lower bound for the strength of associations likely to be observed. However, this manuscript does not provide a comprehensive comparison of methods and completely ignores a whole class of reference free algorithms[23]. Comparing state-of-the-art deconvolution algorithms with this resource indicated that DSA tend to outperform NNLS, CIBERSORT, and dtangle. This suggests that approaches that use reference expression profiles from constitutive cell-types might not be ideal, most likely because a reference dataset suffers from various sample-specific and technical artefacts. In contrast, DSA relies only on the identity of cell-type specific genes, yielding more robust predictions. In the case of the reference datasets used here (mainly derived from single cell data), this variability is likely introduced in the process of isolating cells or measuring gene expression profiles in only the nuclei.

Our results indicate that cell-type deconvolution algorithms can be used to make inferences about cell-type composition at the *sample* and *population* level. For the ROSMAP cohort specifically, this provides support of the inferences that have been made associating proportion of cell-types in the prefrontal cortex of subjects with pathological burden or cognition[3]. We note however that the cell-type proportions derived from IHC and RNA-seq in our study are not from the same tissue section. In fact, the IHC and RNA-seq data have been generated from opposite hemispheres of the same donor and so their comparison will capture differences in this location. Reassuringly, the significant correlations between the IHC and deconvolution estimates suggest that the inter-subject variability is still observable despite of the variability introduced by differences in the tissue location. However, even though the associations between cell-type proportion estimates from IHC and RNA-seq are statistically significant, the correlations are modest in magnitude, and thus our reported correlations are important for providing the context needed to interpret conclusions from these bulk RNA-Seq experiments using post-mortem human brain tissue.

Unfortunately, single-nuclei sequencing data did not appear informative for inferring cell-type proportions. It is well accepted that single-cell sequencing technologies have varying levels of cell-type recovery for different cell-types with substantial optimization often required to detect cell-types of interest[24]. Imaging assays are often considered lossless relative to suspension assays[25] and this potentially offers an explanation for why the IHC cell-type proportions were more correlated with the deconvolution estimates than the proportion calculated by counting cells in the snRNA-seq data. We also saw that MuSiC and BSEQ-sc, methods designed to use single-cell sequencing data to deconvolute bulk expression, did not perform well using the snRNA-seq data as a reference. As both methods performed poorly, we believe this is indicative of the appropriateness of the snRNA-seq data or that more optimization was needed to select appropriate cell-type markers. Regardless, this study indicates that caution should be applied when using either snRNA-seq data to directly calculate cell-type proportions or as a reference set for deconvolution in brain experiments.

Finally, we demonstrated the practical benefits of estimating cell-type proportions in the brain. Alzheimer’s disease and other neurodegenerative diseases have a substantial impact on brain structure and so observations of changes in gene expression in bulk cortical tissue are likely to be masked by changes in cell-type composition. We showed that including cell-type proportion estimates as confounding factors is imperative for reducing false association between gene expression and disease phenotypes. Further, we demonstrated that accounting for this confounding using estimates from cell-type deconvolution algorithms produced significant improvements in cell-type specific eQTL detection by using more accurate marker sets based on multiple genes. These results emphasize that estimates of cell-type proportions generated by deconvolution algorithms have the potential to increase the power, stability and interpretability of gene expression studies using brain tissue.

## Methods and materials

### Sample dissection

During the dissection, one hemisphere is cut into coronal slabs and frozen in a −80°C freezer. The other hemisphere is placed in 4% paraformaldehyde. The frozen middle frontal gyrus (MF) has been used for RNA-seq while the paraffin-embedded fixed tissue from the exact same region, but other hemisphere has been dedicated for immunohistochemistry (IHC). The white matter has been removed from the grey matter of the frozen sample. For both RNA-seq and IHC, only the grey matter has been analyzed.

### IHC image acquisition

Six μm sections of formalin-fixed paraffin embedded tissue have been stained for NeuN (Millipore), GFAP (Dako), Iba1 (Wako), Olig2 (Sigma) and PECAM-1 (Novus biologicals) using antigen retrieval Buffer (Citrate Buffer pH 6.0) for each marker. Sections have been blocked with blocking medium containing 3% BSA and incubated with primary antibodies for overnight at 4oC. Sections have been washed three times with PBS before incubation with Fluorophore-conjugated secondary antibody (Thermofisher) for one hour and coverslipped with anti-fading reagent containing Dapi (P36931, Life technology). Using fluorescence upright microscope (Zeiss Axio), 30 images have been captured in grey matter for each section at magnification x20 with a set exposure time in a systematic zigzag pattern to ensure that all layers of the cortex have been included in quantification.

### IHC image analysis

EBImage[26] was used for all image analysis including background correction, thresholding and segmentation. Automated image analysis was used to identify cell nuclei by DAPI staining and the cells that were positive for a particular cell-type marker. For each participant, proportions were estimated as the average proportion of cell marker positive nuclei across the replicate images. R scripts with the parameters used for estimating the proportions are located on https://github.com/ellispatrick/CortexCellDeconv as well as the corresponding IHC images.

### Defining cell-type markers

Three datasets were used to define marker genes and cell-type reference profiles. Cell-specific reference profiles were collected from single-cell RNA sequencing data (Darmanis)[7] and RNA-seq profiles of purified populations of cells (Zhang)[10] and a set of curated markers from Neuroexpresso[12]. For Darmanis and Zhang, samples were TMM normalized and then voom[27] was used to define marker genes. The markers were selected as the 100 genes with largest fold-change after filtering for genes with false discovery rate less than 0.05. (Performance with respect to varying marker set size is shown in S5 Fig)

### ROSMAP gene expression data

The deconvolution algorithms in this study were applied to the 508 RNA-seq samples from ROSMAP cohort, processed as previously described[3]. Briefly RNA-seq data was adjusted for known technical and biological factors, including age, sex, PMI, PH, and batch by removing the contributions of the factors while maintaining the average expression of each gene. In secondary analysis, we also assessed the deconvolution algorithms on “raw” data with no correction for these confounding factors.

### Description of the deconvolution algorithms

In total, six cell-type deconvolution algorithms were applied to the data; CIBERSORT[15], dtangle[5], DSA[16], NNLS[14], MuSiC[19] and BSEQ-sc[20]. For each of the deconvolution algorithms tested, we used the package provided as part of the primary paper and glmnet [28] was used for NNLS. CIBERSORT, dtangle and NNLS each require both cell-type reference profiles and marker genes while DSA just requires marker genes. For assessing correlations between gene expression and IHC, speakeasy clustering[29], an unsupervised approach, was also evaluated using a set of predefined gene coexpression modules[3] as well as the individual marker genes used in the IHC. As *CD31* wasn’t expressed in the gene expression data, *CD34* was used as the gene marker for endothelial cells instead. See above for the details of the marker set selection approach and https://github.com/ellispatrick/ CortexCellDeconv for R scripts.

### Artificial mixture analysis

To assess the robustness and magnitude of correlations observed in our study, we compared these to an artificial mixture analysis. A single nucleus sequencing dataset from the Allen Brain Atlas[17] was downloaded with 1576 annotated cells from the human lateral geniculate nucleus. Seventy pseudo-bulk expression samples were generated by sampling from these 1576 cells with replacement and averaging the gene expression values of these cells. To imitate the technical and biological noise in a typical sequencing experiment, a dispersion parameter was estimated from the ROSMAP cohort and then this was used to resample read counts from a negative binomial distribution for each pseudo bulk sample. The average profiles of cell-types from the original single nucleus sequencing dataset are then used to deconvolve the new pseudo-bulk dataset and the results are compared to the known proportions.

### Neuronal sub-type analysis

We obtained markers for 7 neuronal sub-types, 2 excitatory and 5 inhibitory, from Darmanis dataset[7]. To assess the robustness of these markers, we compared their magnitude of expression across five major cell-types using Zhang dataset (**S10 Fig**). Given the small number of markers that were truly cell-type specific, we decided to combine markers to two sets: excitatory and inhibitory. Using the DSA algorithm, we estimated the proportion of 4 major cell-types as well as excitatory and inhibitory neurons simultaneously.

### Disease association analysis

We assessed the correlation between gene expression levels and each phenotype in two ways: a univariate model that associates gene expression and a single phenotype, and a multivariate model that includes additional covariates to adjust for cellular heterogeneity. The covariates are the predicted proportions of five major cell-types from the DSA algorithm with Zhang markers. The gene expression data was already adjusted for technical and biological confounding factors as previously described[3]. The scatter plot in Fig 4B reports the p-values for the association between phenotype and expression levels of each gene with the univariate and the multivariate models.

### Cell-type specific eQTL analysis

We used the approach described by Westra and colleagues[21] to identify cell-type specific eQTLs. This approach tests for the statistical significance of a linear interaction model as follows:

y=αg+βc+γ(g×c)

where *y* is a vector of gene expression levels, g is the genotype for the test SNP, *c* is the proportion of test cell-type, and *g x c* is the interaction term between genotype and the proportion of cell-type. The statistical significance of the interaction term, modeled by *γ*, implies the existing of a cell-type-by-genotype effect. As suggested by Westra and colleagues[21], to reduce the burden of multiple testing, only cis-SNPs previously found to be a *cis* xQTL (main effect)[30] using a larger set of ROSMAP samples (N = 508), with a window of 1Mb around TSS, where tested. The cell-type estimates from the DSA algorithm where used. Global false discovery rate (FDR) threshold of 0.1 (correcting for all SNP-gene pairs and cell-types tested) was used to identify significant cell-type-by-genotype eQTLs. The number of cell-type specific eQTLs found here is ~0.02% of the number of eQTLs found previously from the ROSMAP samples[30].

### Ethics statement

All participants signed an informed consent approved by the Institutional Review Board of RUSH University.

## Supporting information

S1 Table Table presents the demographic information of the subjects for whom IHC data was generated. (XLSX)

S1 Fig Association between cell-type proportions and Alzheimer’s disease. Cell-type proportions calculated from IHC data are compared between individuals with and without Alzheimer’s Disease (AD).
(TIF)

S2 Fig Correlation between IHC estimates and expression level of gene modules. Each dot depicts an individual. Our previous study defined a set of modules with gene members that were enriched for each of the five cell-types examined (Mostafavi and Gaiteri et al., Nat Neur 2018): the average expression of each of these modules (across genes) represents a relative score for each individuals that can serve as a proxy for proportion of the corresponding cell-type. The module average expression is shown on the x-axis and the IHC-based proportions are shown on the y-axis.
(TIF)

S3 Fig Correlation between predicted and estimated proportions. Scatter plots show the inferred and measured proportions for five cell-types across four different methods.
(TIF)

S4 Fig Correlation of different deconvolution methods. Plots show the pairwise correlation between pairs of deconvolution methods using the Zhang markers, assessed based on 508 samples.
(TIF)

S5 Fig Assessing varied sources of marker gene sets. Figure shows the performance of (A) DSA and (B) dtangle methods, based on different sources for marker gene set selection: scRNA-seq based markers (Darmanis), human cell sorted (Zhang) and mouse microarray and ISH (NeuroExpresso). Y-axis shows the correlation between the prediction and IHC across 70 ROSMAP samples. (C) Figure shows the correlation between all 4 methods and single gene markers, as inferred using “Darmanis” markers, with measured IHC data.
(TIF)

S6 Fig Accuracy of deconvolution on raw vs adjusted data. For data from post-mortem brain, in addition to RNA integrity number (RIN), other technical factors such as PH and post-mortem interval (PMI) are known to have a major impact on the estimated gene expression levels. To assess whether correction for these variables impacts the accuracy of cell-type proportions, we applied deconvolution algorithms on “raw” and “adjusted” data (see Methods). Figure shows the correlation between cell-type proportions inferred from four different deconvolution algorithms and the measured IHC proportions.
(TIF)

S7 Fig Quantifying the upper bound of correlation coefficient using artificial mixtures. Figure shows the achievable range of correlation coefficient on the simulated experiment. Artificial bulk gene expression data was created for deconvolution by sampling with replacement from a population of 1576 annotated cells from the human lateral geniculate nucleus and averaging. After a dispersion parameter was estimated from the ROSMAP cohort, these average profiles were resampled from a negative binomial distribution to emulate technical and biological noise. These pseudo-bulk profiles were then deconvolved using the original single nucleus sequencing data and the estimated proportions were compared to the truth.
(TIF)

S8 Fig Accuracy of predicted proportions with variable marker gene set size. (A) Population-level range of prediction of absolute proportions with variable size of marker gene sets based on Darmanis markers. (B) Correlation between prediction of cell-type proportions with variable sizes of marker gene sets. Differential expression analysis using single cell data was used to define marker gene sets.
(TIF)

S9 Fig Proportion of nuclei assigned to various cell-types according to Dronc-Seq single-cell data from cortex and hippocampus. Figure summarizes the proportion of nuclei assigned to various cell-types (Habib et al., Nature Methods 2017). A: astrocytes; E: endothelial cells; M: microglia; N1,N2,N3,N4: different neuronal populations; O: oligodendrocyte; OP: oligodendrocyte progenitor cells.
(TIF)

S10 Fig Quantifying accuracy of inferred absolute proportions across individuals. Mean squared error (MSE) quantified across 70 individuals, using (A) Zhang and (B) Darmanis markers as input to deconvolution algorithms. STD refers to the standard deviation of the IHC measurements for each cell-types. (C-D) Figures show the significance (log10 pvalues) for the estimated MSE, as assessed by permutation tests using 10000 permutations, where Zhang (C) and Darmanis (D) markers are used.
(TIF)

S11 Fig Correlation of MuSiC and BSEQ-sc proportions with IHC proportions. The Spearman correlation coefficient between IHC derived cell-type proportions and six deconvolution algorithms. Included here are two methods, MuSic and BSEQ-sc. Both of these methods are designed to use single-cell sequencing data as a reference set to deconvolute bulk expression. Here they are using the ROSMAP snRNA-seq data as a reference to deconvolute the ROSMAP bulk gene expression data.
(TIF)

S12 Fig Heatmaps of cell-type markers from the Darmanis dataset. In order to assess the performance of deconvolution of cell-type subsets we obtained markers of excitatory and inhibitory, neurons from reported by Darmanis et al. These sub-type markers were not specific to neurons (A) and so they were filtered to those that were specifically highly expressed in neurons in the Darmanis data (B).
(TIF)
