# Supplementary figures and images for: Deconvolving the contributions of cell-type heterogeneity on cortical gene expression
Source: PLoS Comput Biol. 2020 Aug 17;16(8):e1008120. doi: 10.1371/journal.pcbi.1008120 (PMC7451979; doi:10.1371/journal.pcbi.1008120)

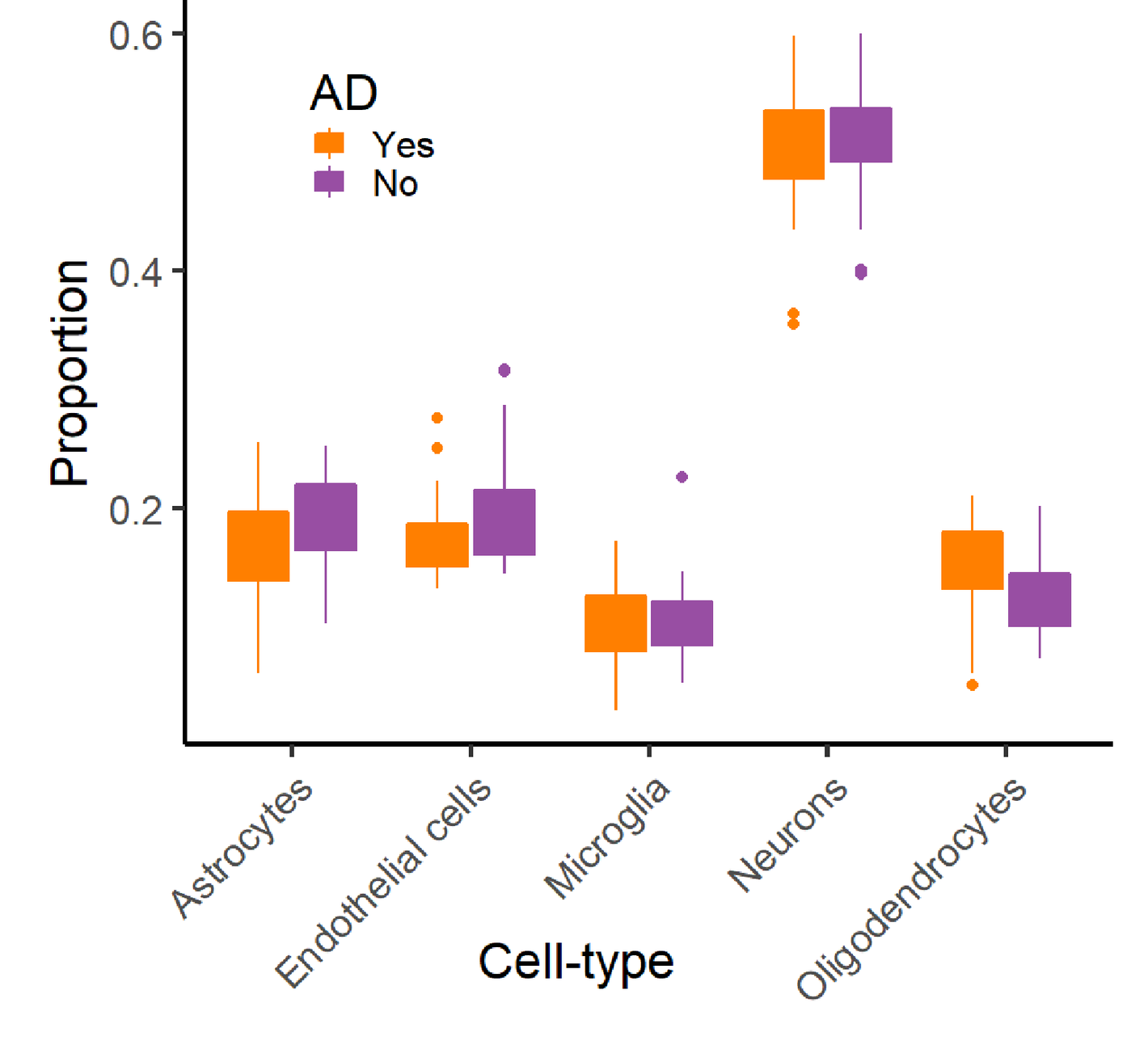

Supplement: S1 Fig — Cell-type proportions calculated from IHC data are compared between individuals with and without Alzheimer’s Disease (AD). (TIF) [file pcbi.1008120.s002.tif]

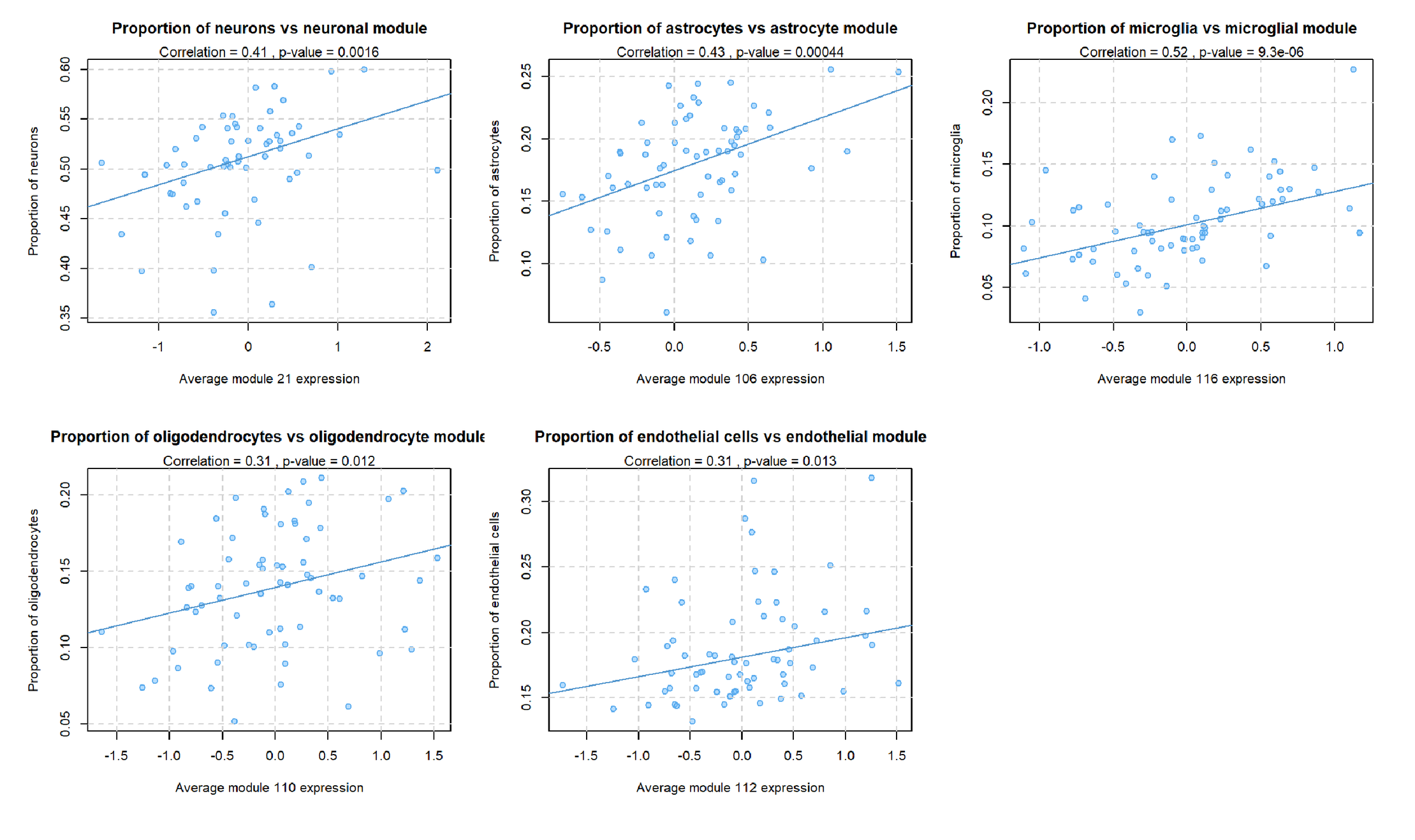

Supplement: S2 Fig — Each dot depicts an individual. Our previous study defined a set of modules with gene members that were enriched for each of the five cell-types examined (Mostafavi and Gaiteri et al., Nat Neur 2018): the average expression of each of these modules (across genes) represents a relative score for each individuals that can serve as a proxy for proportion of the corresponding cell-type. The module average expression is shown on the x-axis and the IHC-based proportions are shown on the y-axis. (TIF) [file pcbi.1008120.s003.tif]

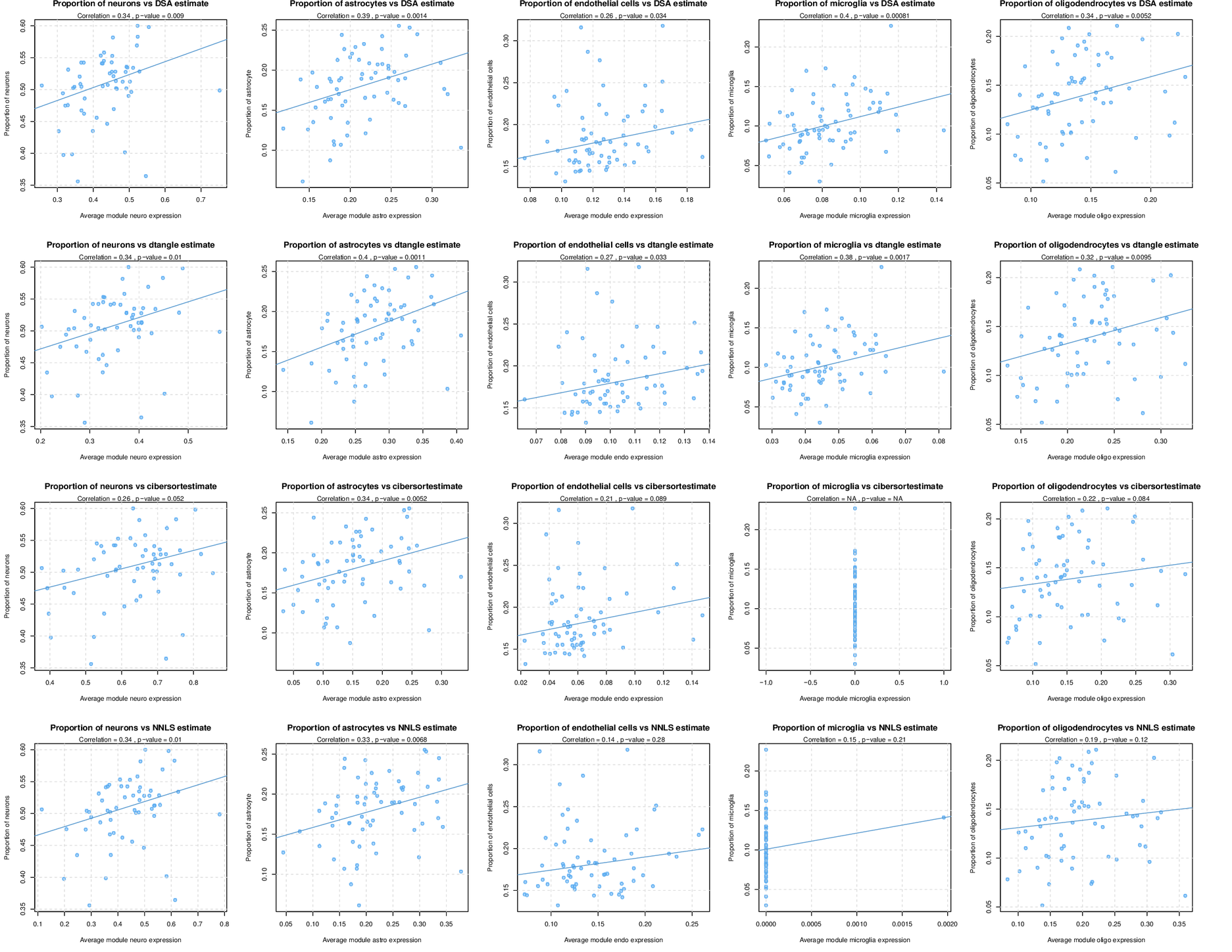

Supplement: S3 Fig — Scatter plots show the inferred and measured proportions for five cell-types across four different methods. (TIF) [file pcbi.1008120.s004.tif]

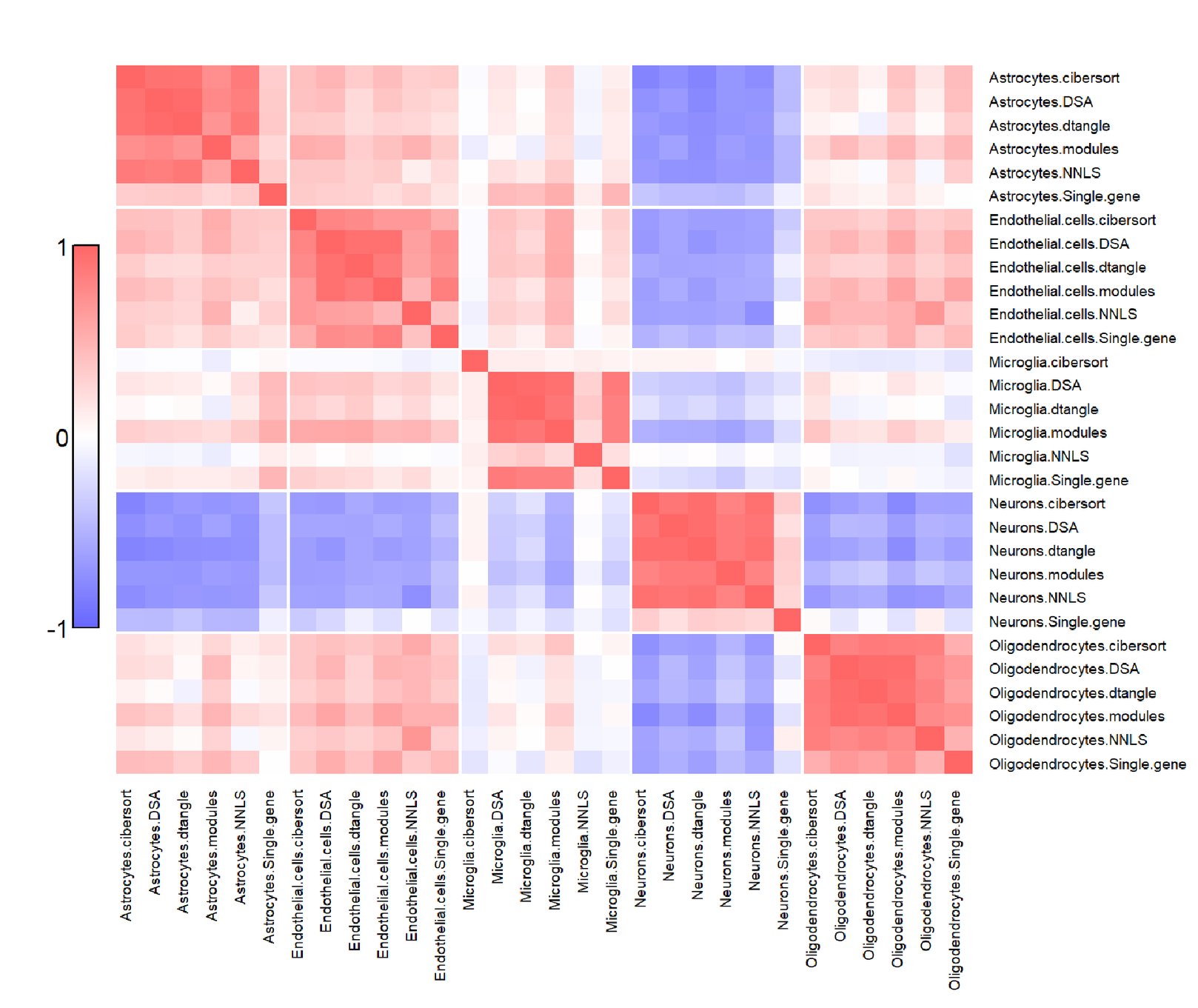

Supplement: S4 Fig — Plots show the pairwise correlation between pairs of deconvolution methods using the Zhang markers, assessed based on 508 samples. (TIF) [file pcbi.1008120.s005.tif]

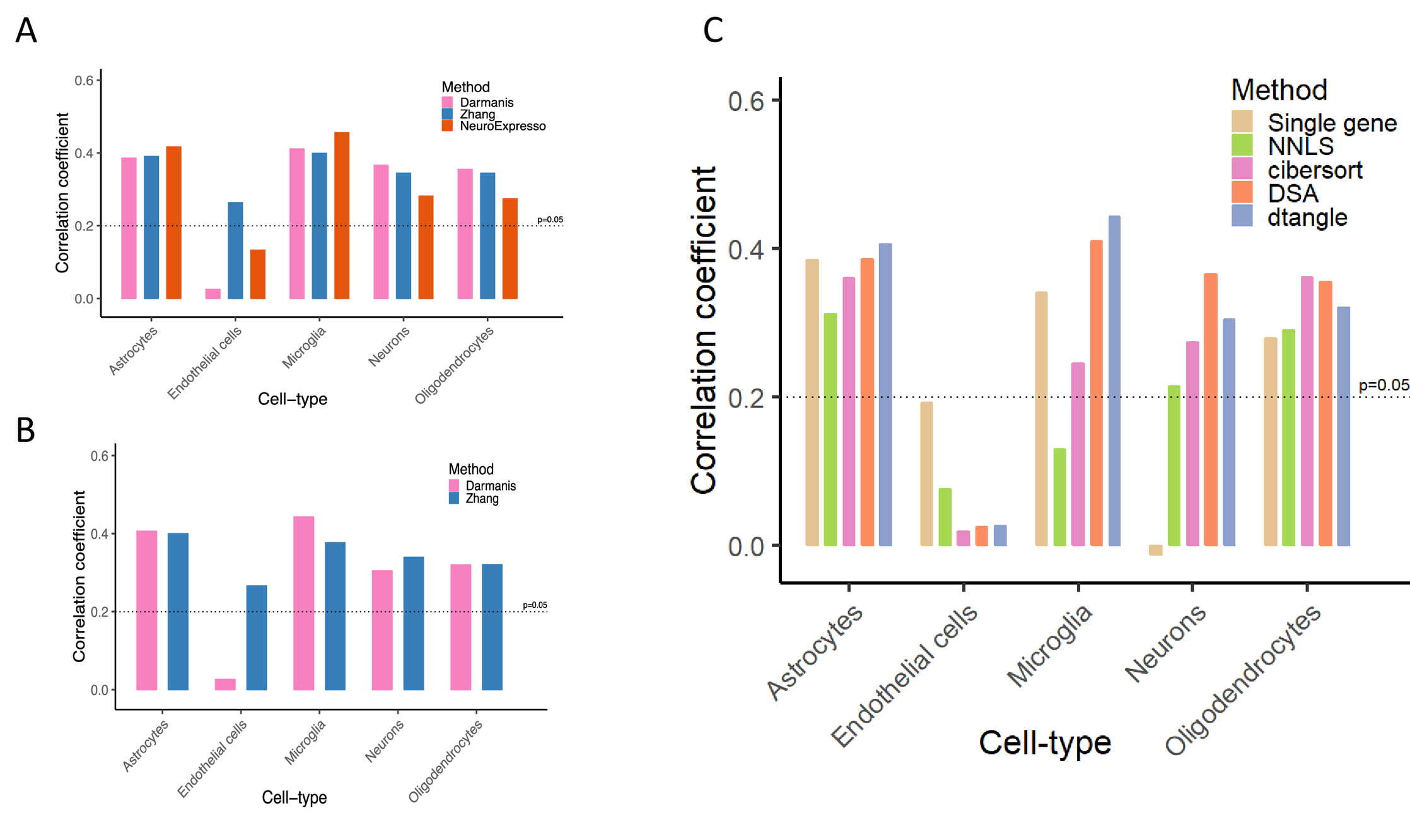

Supplement: S5 Fig — Figure shows the performance of (A) DSA and (B) dtangle methods, based on different sources for marker gene set selection: scRNA-seq based markers (Darmanis), human cell sorted (Zhang) and mouse microarray and ISH (NeuroExpresso). Y-axis shows the correlation between the prediction and IHC across 70 ROSMAP samples. (C) Figure shows the correlation between all 4 methods and single gene markers, as inferred using “Darmanis” markers, with measured IHC data. (TIF) [file pcbi.1008120.s006.tif]

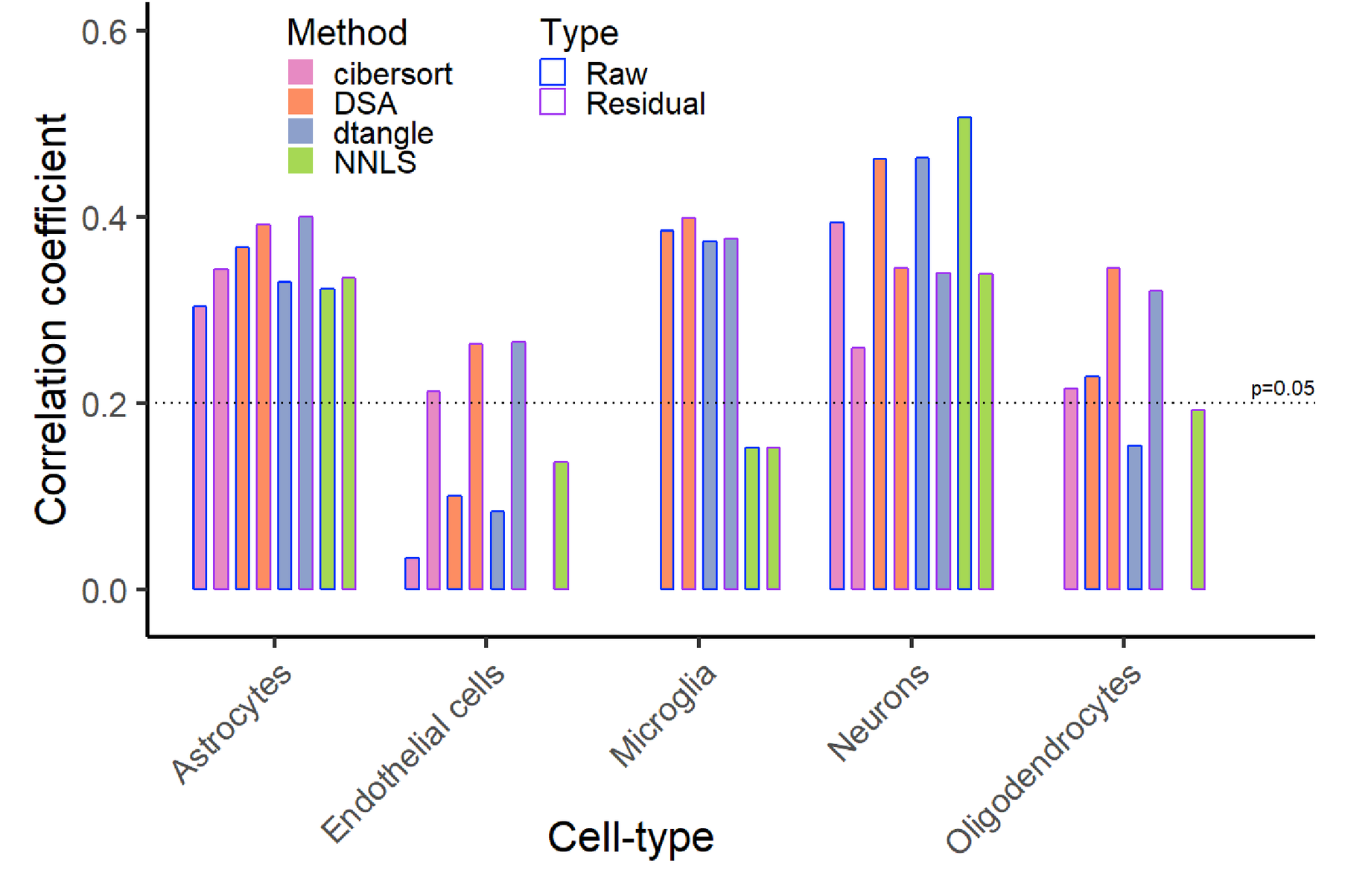

Supplement: S6 Fig — For data from post-mortem brain, in addition to RNA integrity number (RIN), other technical factors such as PH and post-mortem interval (PMI) are known to have a major impact on the estimated gene expression levels. To assess whether correction for these variables impacts the accuracy of cell-type proportions, we applied deconvolution algorithms on “raw” and “adjusted” data (see Methods). Figure shows the correlation between cell-type proportions inferred from four different deconvolution algorithms and the measured IHC proportions. (TIF) [file pcbi.1008120.s007.tif]

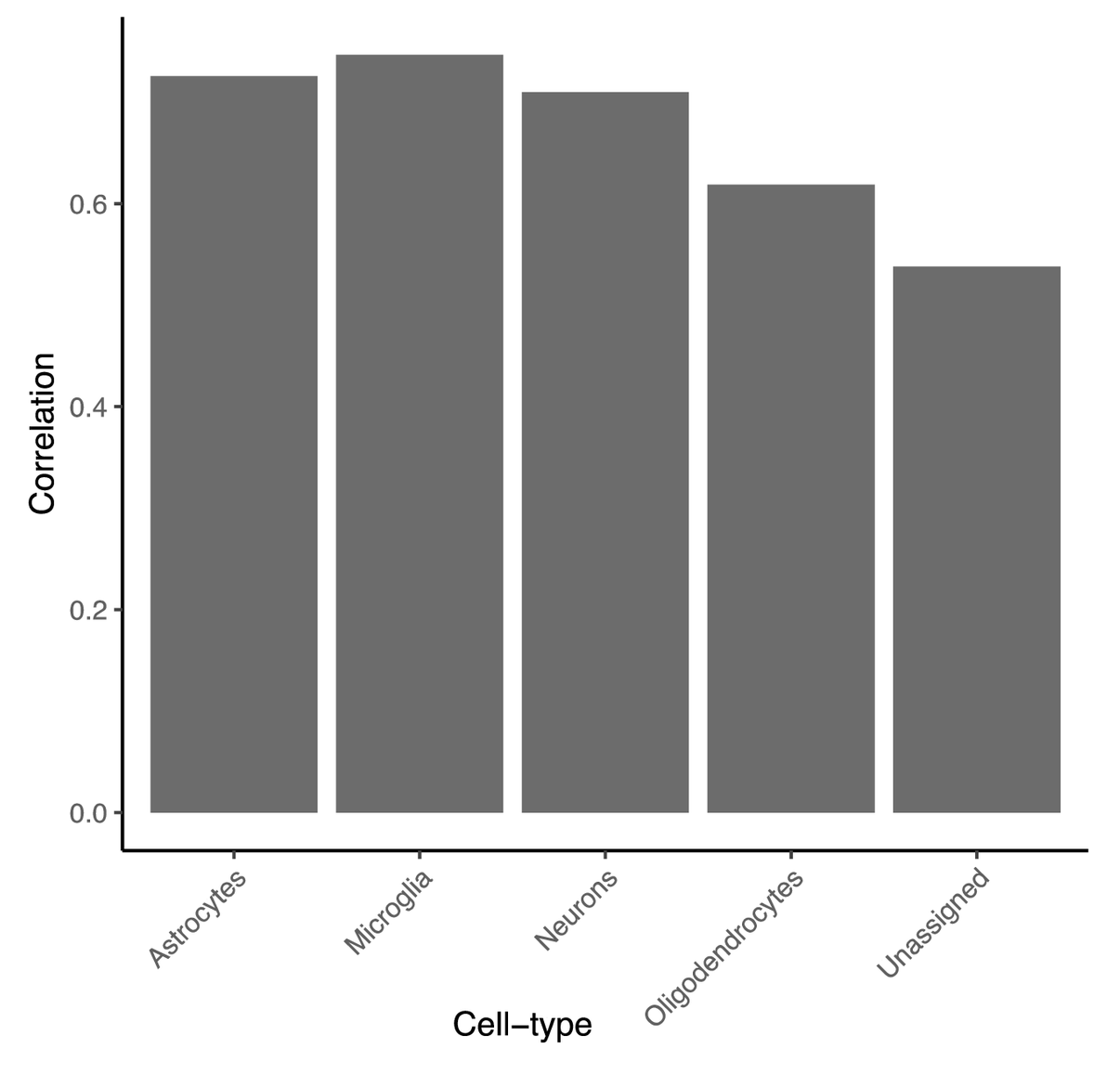

Supplement: S7 Fig — Figure shows the achievable range of correlation coefficient on the simulated experiment. Artificial bulk gene expression data was created for deconvolution by sampling with replacement from a population of 1576 annotated cells from the human lateral geniculate nucleus and averaging. After a dispersion parameter was estimated from the ROSMAP cohort, these average profiles were resampled from a negative binomial distribution to emulate technical and biological noise. These pseudo-bulk profiles were then deconvolved using the original single nucleus sequencing data and the estimated proportions were compared to the truth. (TIF) [file pcbi.1008120.s008.tif]

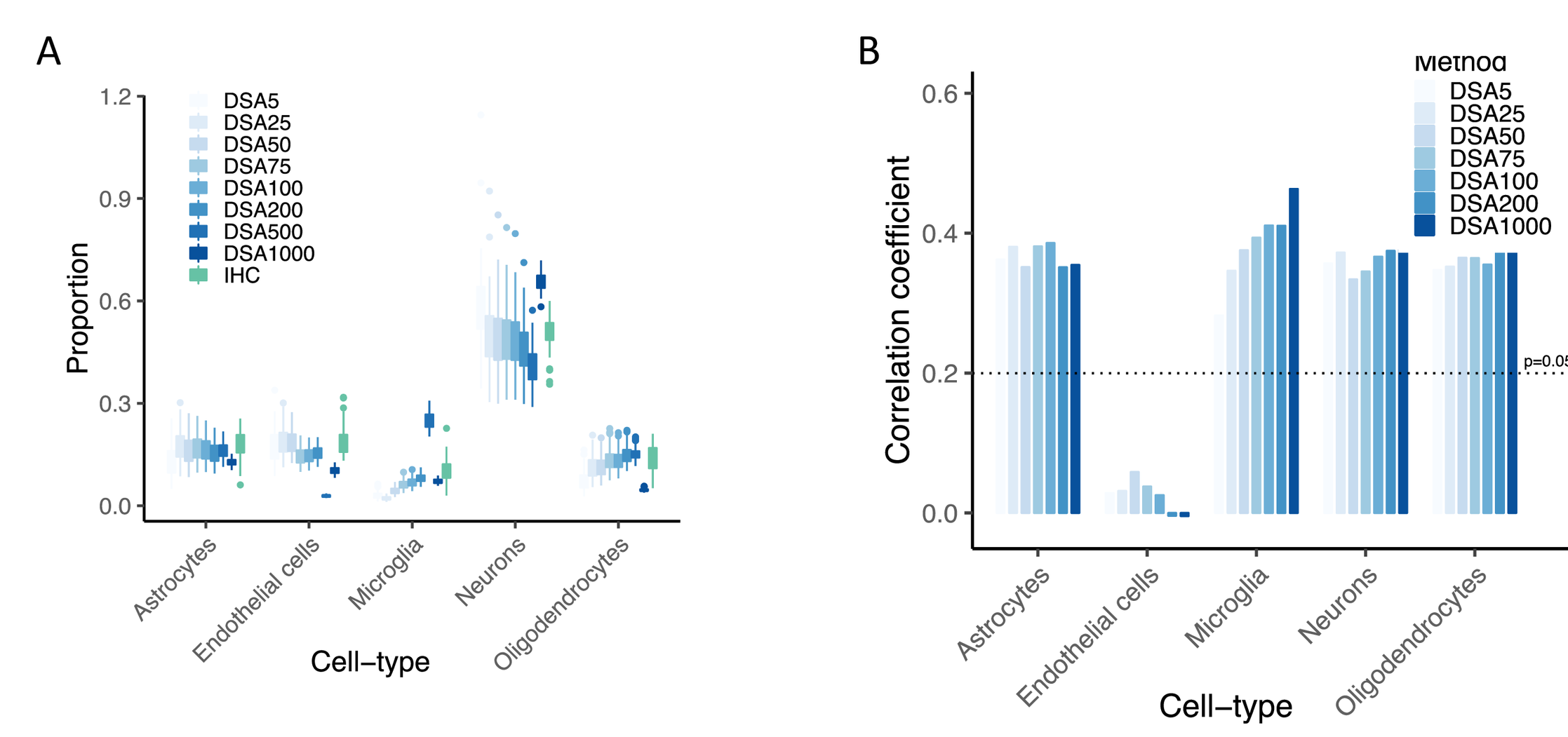

Supplement: S8 Fig — (A) Population-level range of prediction of absolute proportions with variable size of marker gene sets based on Darmanis markers. (B) Correlation between prediction of cell-type proportions with variable sizes of marker gene sets. Differential expression analysis using single cell data was used to define marker gene sets. (TIF) [file pcbi.1008120.s009.tif]

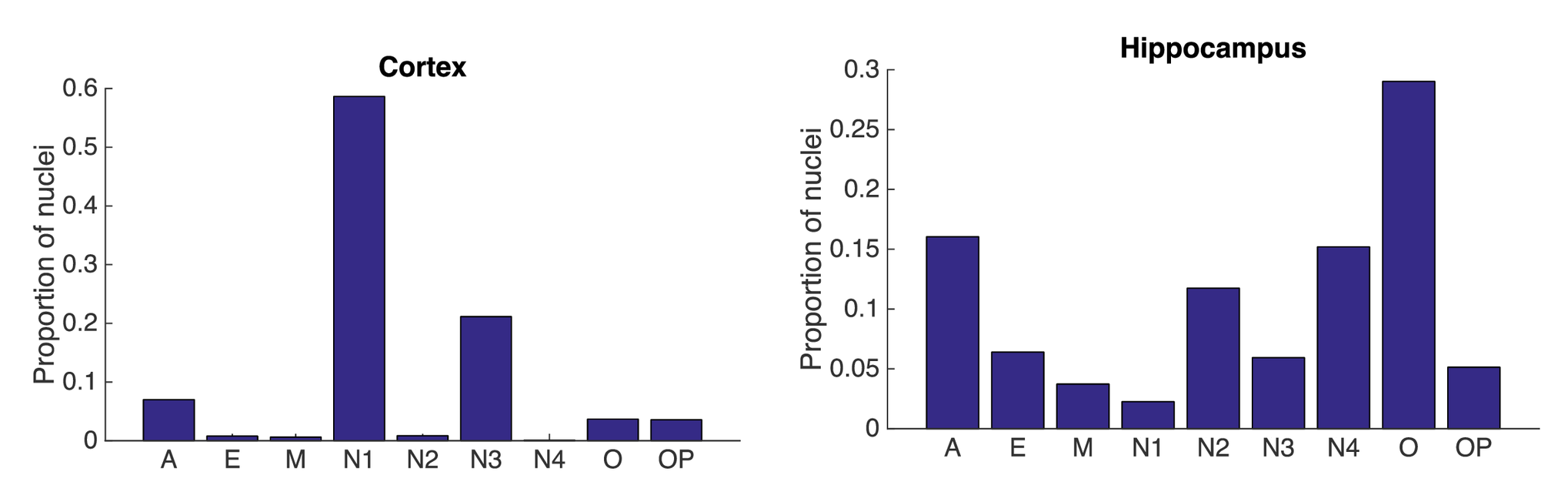

Supplement: S9 Fig — Figure summarizes the proportion of nuclei assigned to various cell-types (Habib et al., Nature Methods 2017). A: astrocytes; E: endothelial cells; M: microglia; N1,N2,N3,N4: different neuronal populations; O: oligodendrocyte; OP: oligodendrocyte progenitor cells. (TIF) [file pcbi.1008120.s010.tif]

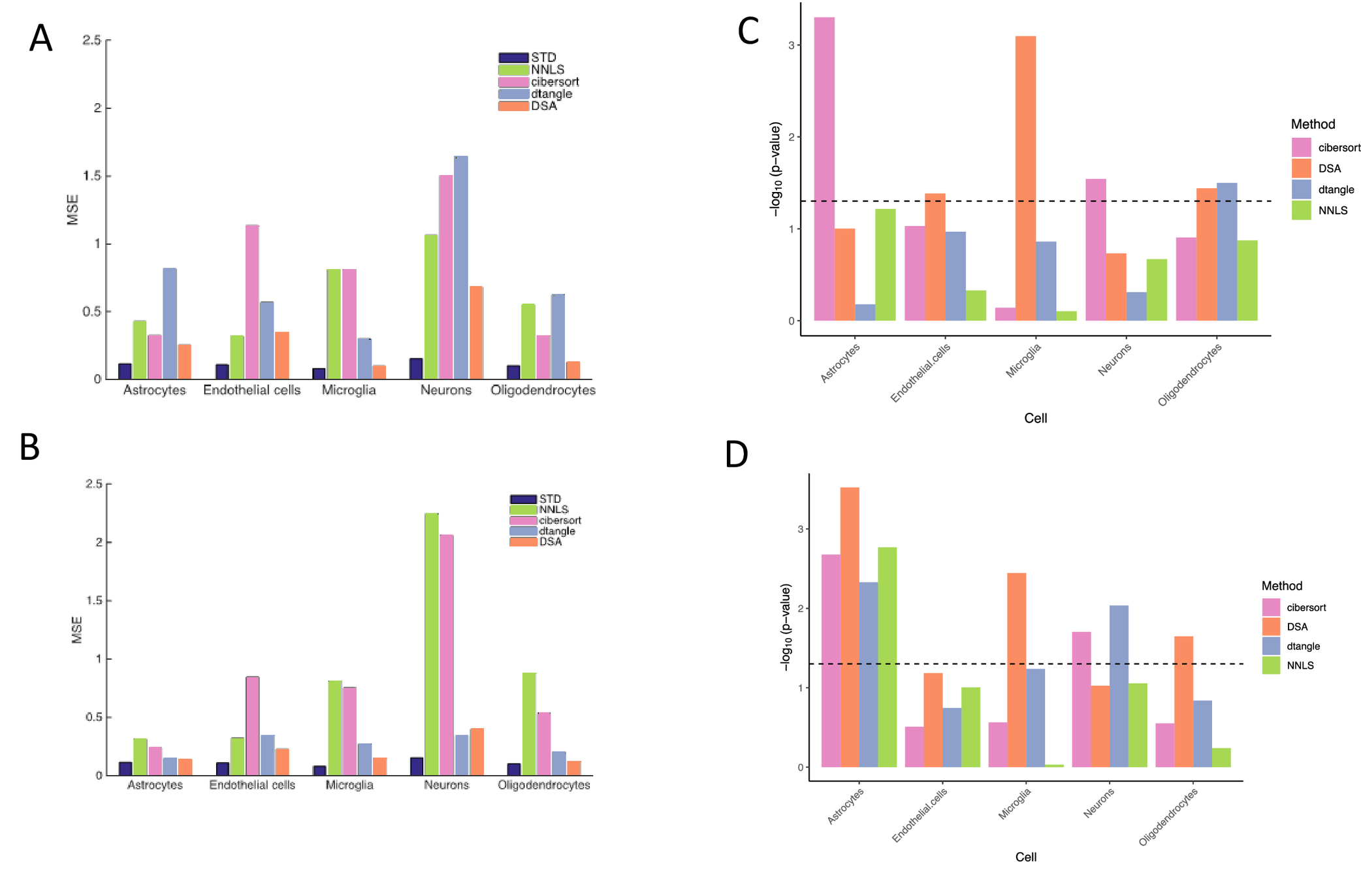

Supplement: S10 Fig — Mean squared error (MSE) quantified across 70 individuals, using (A) Zhang and (B) Darmanis markers as input to deconvolution algorithms. STD refers to the standard deviation of the IHC measurements for each cell-types. (C-D) Figures show the significance (log10 pvalues) for the estimated MSE, as assessed by permutation tests using 10000 permutations, where Zhang (C) and Darmanis (D) markers are used. (TIF) [file pcbi.1008120.s011.tif]

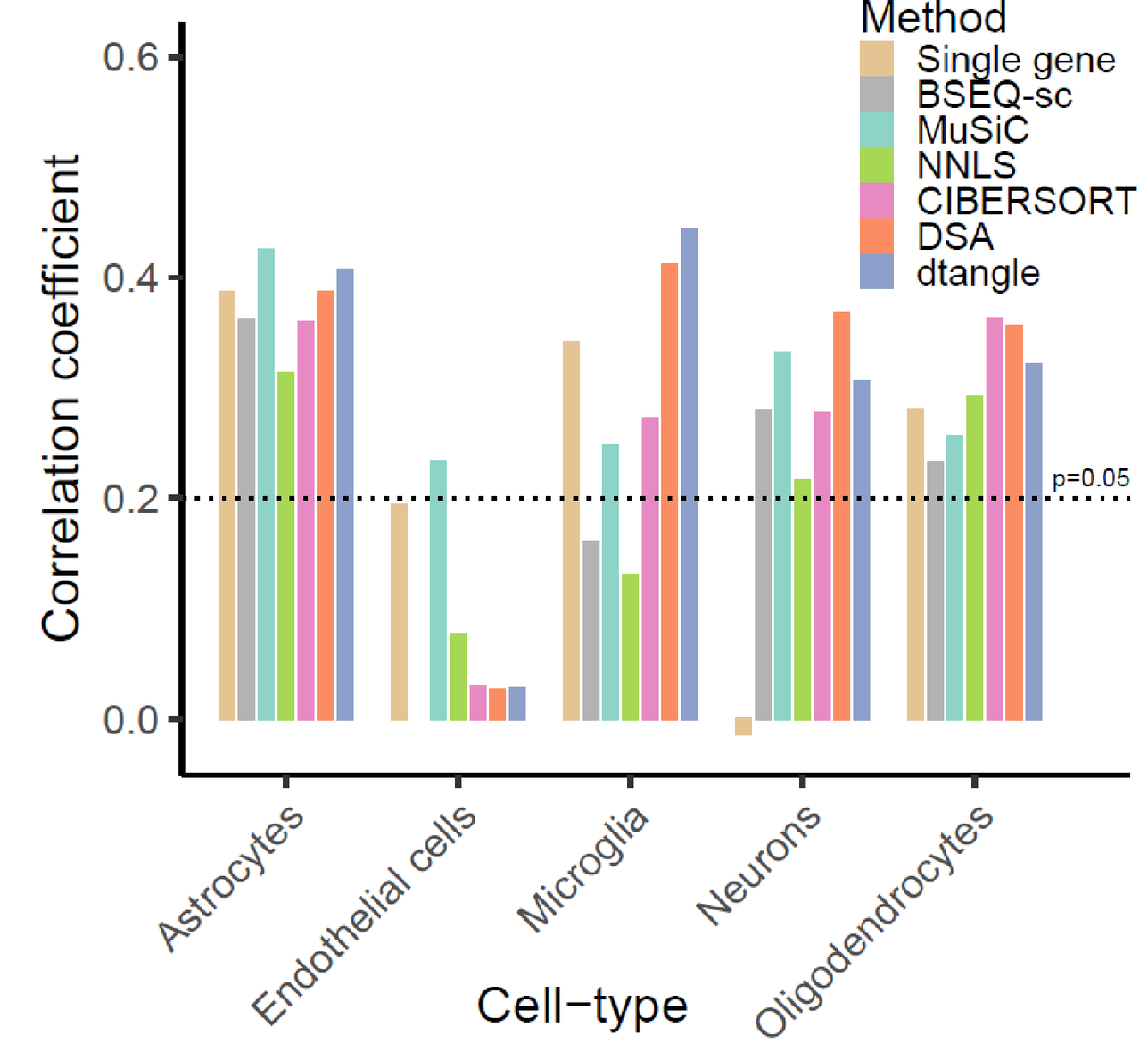

Supplement: S11 Fig — The Spearman correlation coefficient between IHC derived cell-type proportions and six deconvolution algorithms. Included here are two methods, MuSic and BSEQ-sc. Both of these methods are designed to use single-cell sequencing data as a reference set to deconvolute bulk expression. Here they are using the ROSMAP snRNA-seq data as a reference to deconvolute the ROSMAP bulk gene expression data. (TIF) [file pcbi.1008120.s012.tif]

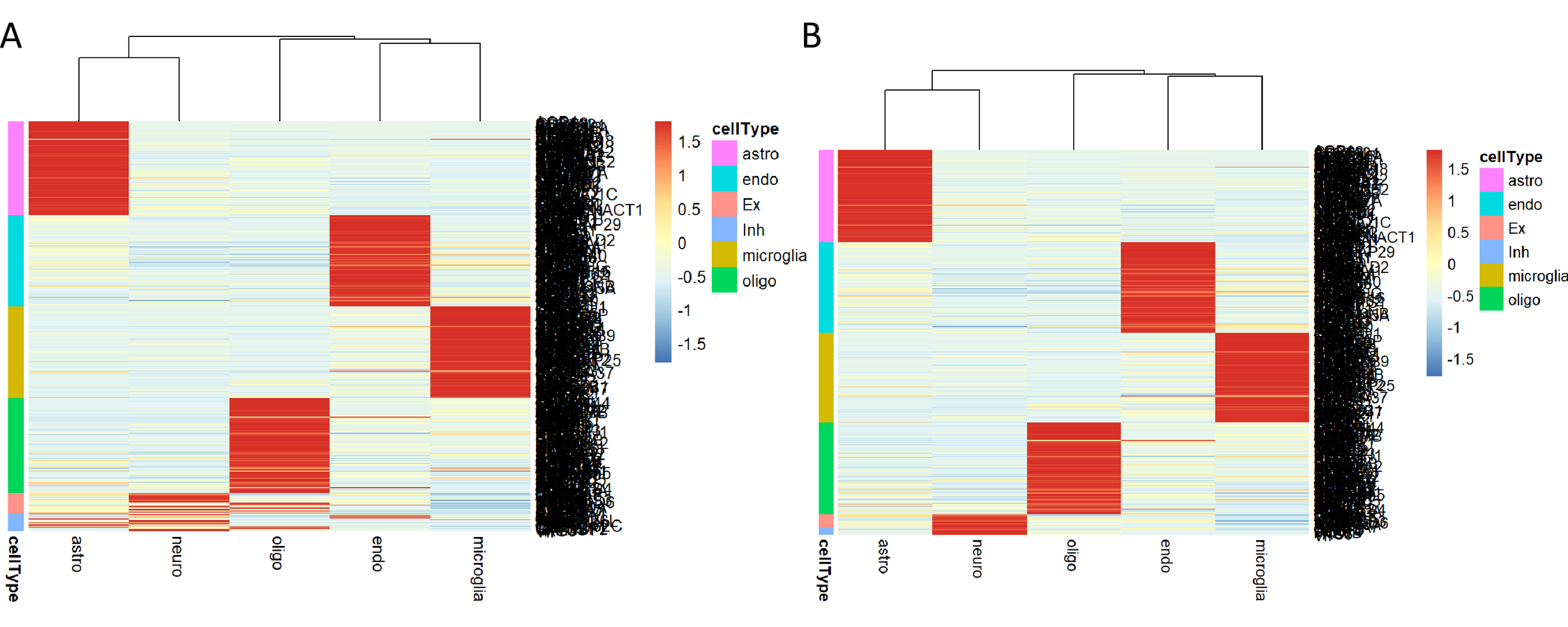

Supplement: S12 Fig — In order to assess the performance of deconvolution of cell-type subsets we obtained markers of excitatory and inhibitory, neurons from reported by Darmanis et al. These sub-type markers were not specific to neurons (A) and so they were filtered to those that were specifically highly expressed in neurons in the Darmanis data (B). (TIF) [file pcbi.1008120.s013.tif]
